# Association Between the Corrected QT Interval and Carotid Artery Intima-Media Thickness in Obese Children

**DOI:** 10.4274/jcrpe.v2i1.21

**Published:** 2010-12-08

**Authors:** Ayla Güven, Tolga Özgen, Olcay Güngör, Murat Aydın, Kemal Baysal

**Affiliations:** 1 Göztepe Educational and Research Hospital, Clinics of Pediatric Endocrine, İstanbul, Turkey; 2 Ondokuz Mayıs University Medical Faculty, Department of Pediatric Endocrinology, Samsun, Turkey; 3 Ondokuz Mayıs University Medical Faculty, Department of Pediatric Cardiology, Samsun, Turkey; +90 532 238 03 00+90 216 566 40 23aylaguven@yahoo.comGöztepe Educational and Research Hospital, Clinics of Pediatric Endocrine Kadıköy, İstanbul, Turkey

**Keywords:** obesity, QTc, carotid artery intima-media thickness

## Abstract

**Objective**: Sudden death has been reported in asymptomatic obese adults and the mechanism is unclear. In recent years, obesity has shown a dramatic increase in children and this enhances the risk factors for the development of cardiovascular disease. The aim of this study was to investigate whether there is repolarization abnormality and any potential risk factor such as increase in intima-media thickness (IMT) of carotid artery for corrected QT (QTc) prolongation among obese children.

**Methods**: A total of 60 obese children, 30 of which had features of metabolic syndrome (MS), and 23 age-matched controls were included in the study. QTc interval was calculated at rest. The IMT of both common carotid arteries (CCA) was measured. The relationship between QTc, IMT of right and left CCA and insulin sensitivity indices were evaluated in the study group.

**Results**: The QTc interval of the children with simple exogenous obesity (SEO) were longer than in the controls (p=0.024). The IMT of both carotid arteries of the obese girls and boys with and without MS were higher than the controls (p=<0.001). The QTc was significantly affected by the parameters pertaining to the right carotid artery IMT, to chronologic age and HDL-C.

**Conclusion**: Since obesity may cause subclinical atherosclerotic disease regardless of sex, obese children must be followed closely for early cardiovascular problems.

**Conflict of interest:**None declared.

## INTRODUCTION

The prevalence of childhood obesity is increasing rapidly, particularly in developed countries. It has also been shown that increased frequency of obesity in children parallels the increase in risk factors for the development of cardiovascular diseases (1, 2, 3). Sudden death has been reported in obese adults who have no apparent heart abnormalities; the mechanism is unclear (4). Results of studies concerning prolongation of the corrected QT (QTc) in obese adults are controversial (5, 6, 7). It has been shown that adults with metabolic syndrome (MS) have higher values of QTc-min and QTc-max (8). The QT dispersion (QTc-d) has been suggested as a physiological variability of regional ventricular repolarization. Physiological hyperinsulinemia acutely prolongs ventricular repolarization independent of insulin sensitivity (9). QTc prolongation and increased QTc-d have been reported in children with type 1 diabetes, but the mechanism remains unknown (10). Also, it has been shown that QTc interval prolongation in adults with type 2 diabetes is associated with ischemic heart disease (11), and that patients with prolonged QTc interval have higher mortality from cardiovascular disease (12).

Obese children have endothelial dysfunction associated with fatty streaks and fibrous plaques (13, 14). They are also at increased risk for coronary artery calcification and coronary heart disease in adulthood (15, 16). Carotid artery intima-media thickening (IMT) provides an indirect assessment of coronary disease. In adults, there is a strong association between coronary vascular disease and carotid artery IMT (17, 18). Carotid artery IMT also progresses faster in the presence of coronary artery disease (19). The echocardiographic measurement of carotid artery IMT is useful in showing generalized atherosclerosis (18, 20). A positive correlation was found between carotid artery IMT and prolonged QTc (21). 

The aim of the present study was to investigate whether there is repolarization abnormality in obese children. The potential risk factors for QTc prolongation in these children have also been investigated.

## METHODS

A total of 60 asymptomatic obese children and adolescents [13.3±2.4 (range 8.2-17.7) years] and 23 age-matched controls [12.94±2.43 (6.1-16.2) years] were included in the study. Patients followed in the endocrinology outpatient clinic for short stature and who were free of any cardiac symptom constituted the control group. There were no children in the study or control groups who were on any medication known to modify the QT interval. 

The height and weight of all individuals were recorded between 09.00 and 12.00 am and their body mass indices (BMI) were calculated. All subjects in the study group had BMI values exceeding the 95th percentile for age and sex, and all were classified as obese (22).

Blood pressure was measured in sitting position in the right arm with a mercury sphygmomanometer following a 15-minute rest period. Mean arterial pressure (MAP) was calculated with the following formula (23). 

Diastolic blood pressure (DBP)+[systolic blood pressure (SBP)-DBP)]/3 

All subjects having a SBP and/or DBP higher than the 95^th^ percentile for age and sex were classified as hypertensive (24).

After routine physical examination, a resting 12-lead electrocardiogram with a rhythm strip was recorded during the same time interval (09.00-12.00 am). This standard 12-lead electrocardiogram was performed with a paper speed of 25 mm/sec and amplitude of 10 mm/mV (Schiller AT-2 plus, Switzerland); 5 consecutive beats were evaluated on lead II rhythm strip. The QT interval was taken as the value from the beginning of the QRS complex to the end of the down slope of the T wave (crossing of the isoelectric line). The QTc for the previous cardiac R-R cycle length was calculated according to Bazett’s formula (25). The QTc-d was calculated as the difference between the longest and the shortest QTc interval, measured in each of the 12 electrocardiographic leads. Two physicians measured all the intervals; to minimize interphysician variability, the mean of the two calculations has been used for analysis. A QTc >440 ms was considered abnormally prolonged, according to the commonly used criteria (26). 

Echocardiographic studies were performed with ATL HDI 3000 CV system (ATL, Germany). Subjects were examined in a supine position. A longitudinal view of the distal common carotid artery was obtained from the suprasternal notch using a 3-5 MHz sector transducer. The IMT measurements were made in both carotid arteries at about 2 cm after the bifurcation. All measurements wererepeated twice by the same observer. 

Fasting (≥10-hours) blood samples were obtained from all subjects, in the study population for measuring the levels of glucose, insulin, total cholesterol (TC), triglyceride (TG) and high-density lipoprotein-cholesterol (HDL-C). Low-density lipoprotein (LDL-C) concentration was calculated by using the Friedewald equation [LDL-C=TC- (HDL-C –(TG/5)] (27). After a high- carbohydrate diet (300 g) for 3 days and an 10-hour overnight fasting, oral glucose tolerance test (OGTT) was performed in all obese children (1.75 g/kg body weight, maximum 75 g glucose) and the venous blood glucose and insulin levels were determined at 0, 30, 60, 90 and 120 minutes. OGTT was not performed in the control group for ethical reasons. Homeostatic model assessment (HOMA-IR) was calculated as the product of the fasting plasma insulin levels (mIU/mL) and fasting plasma glucose levels (mmol/L), divided by 22.5 (28). Fasting glucose/insulin ratio (FGIR) was calculated from the fasting plasma glucose and serum insulin levels. Quantitative insulin sensitivity check index (QUICKI) was determined by the formula (29). 

1/[Log fasting insulin (mU/mL)+Log fasting glucose (mg/dL)] 

Serum glucose was measured by enzymatic-spectrophotometric glucose oxidase method (Roche Diagnostics, USA). Insulin was assayed by chemiluminescence method using the commercial kit from DPC (Diagnostic Products Corporation, USA). Lipid parameters were determined by enzymatic methods using commercial kits. All analyses were performed by using standard instruments (Roche/Hitachi 917, Japan). Serum levels of TC, TG and HDL-C were also measured by the Roche Diagnostics system.

A global risk factor cluster score was assigned to each participant on the basis of presence or absence of up to 5 age-adjusted risk factors: obesity, high serum fasting insulin level (>15 mIU/mL), high TG level (>100 mg/dL), high LDL-C level (>130 mg/dL), low HDL-C level (<40 mg/dL) and high blood pressure (either SBP or DBP). MS in children was defined as having at least three of the defined risk criteria (30, 31). According to these criteria, the obese children were divided into two groups as MS and simple exogenous obese (SEO).

**Statistical Analysis**

Statistical analysis was performed using SPSS 10 Statistical Software Package (SPSS Inc., Chicago, IL, USA). Auxological data, SBP, DBP, MAP and hormonal results were expressed as mean±SD, unless otherwise stated. Normality of the data was evaluated by the “Kolmogorov Smirnov test”. Differences in the parameters between obese groups were investigated using the independent samples t-test. One-way analysis of variance followed by the least significant difference post-hoc test (LSD) was used to assess the significance of the differences in QTc, QTc-d, right and left carotid IMT in the obese and control groups. Pearson’s r and Kendall’s tau-b correlation coefficients were used for the analysis of intercorrelations among the investigated parameters. Linear regression analysis was applied to carotid artery IMT (mm) and QTc (msc) data. A p<0.05 was considered statistically significant.

**Ethics**

Approval for the study was obtained from the Institutional Review Board of the Ondokuz Mayıs University Faculty of Medicine. The purpose of the study was explained to all subjects before participation and written informed consent was obtained from the parents of the study and the control groups.

## RESULTS

Table 1 shows the anthropometric (weight, height, BMI) and clinical characteristics of the study population. There were no differences between the two sexes in clinical and laboratory parameters. 

The values of systolic blood pressure, HOMA-IR, fasting insulin, glucose at 120 minutes and insulin at 120 minutes were higher in the MS group than in the SEO. The QUICKI value of the MS group was lower than that of the SEO group and the controls (Table 1 and 2). Twenty seven (18 female) obese children had hypertension. Twenty (81%) of these children had findings consistent with other criteria of MS. 

QTc duration ranged from 0.337 to 0.427 ms in MS group; from 0.347 to 0.455 in SEO group. The QTc of the obese patients was longer than the controls (p=0.024) (Table 3). Also, five of the SEO girls had long QTc (16.6%). QUICKI and FGIR were negatively correlated with QTc (r=-0.268, p=0.041) in the obese children.

The IMT of the study population was evaluated after adjustment for BMI. The IMT measurements of both carotid arteries in the obese groups were higher than the controls (p<0.001) (Table 3). 

**Figure 1:**
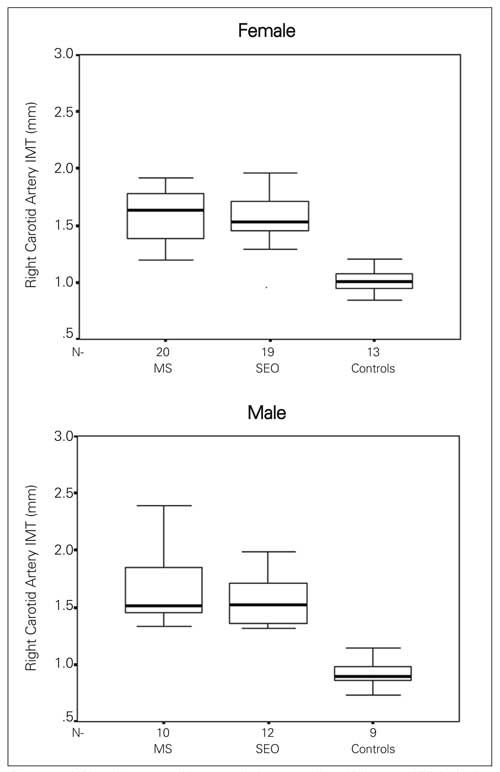
The right carotid artery intima-media thickness (IMT) of the obese groups and controls *Median, 25^th^-75^th^ centiles, minimum and maximum values are denoted.

**Figure 3:**
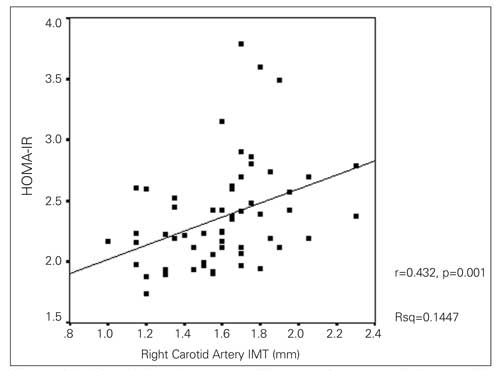
Correlation between right carotis artery intima-media thickness (IMT) and hoemostatic model assessment for insulin resistance (HOMA-IR)

Both right (RCA) and left carotid artery (LCA) IMT values were higher in both male and female obese children when compared with controls (Figure 1 and 2). The minimum and maximum values of the RCA-IMT in the study group were 1.19-2.47 mm in obese children with MS, 1.29-2.65 mm in the SEO group and 0.73-1.21 mm in the control group. The minimum and maximum values of LCA-IMT in study groups were 1.22-2.52 mm in the obese with MS, 1.31-2.65 mm in the SEO group and 0.75-1.23 mm in the control group. The youngest child in the study group was a 8 7/12 year-old girl, who had MS and had higher LCA and RCA-IMT values than the control group (RCA- IMT was 1.30 mm, and LCA-IMT 1.33 mm). HOMA-IR showed a statistically significant correlation with RCA-IMT (r=0.432, p=0.001) (Figure 3) (r=0.276, p=0.033) in the obese group. 

QTc was negatively correlated with QUICKI (r=-0.268, p=0.041) and FGIR (r=-0.268, p=0.041) in the obese children. QTc was significantly affected by RCA-IMT, chronologic age and HDL-C levels, according to the results of stepwise linear regression analysis (Table 4). RCA-IMT was the statistically significant determinant for LCA-IMT according to the multivariate linear regression analysis in both obese groups. BMI and LCA-IMT were the statistically significant determinants for RCA-IMT according to the multivariate linear regression analysis (Table 5).

**2:**
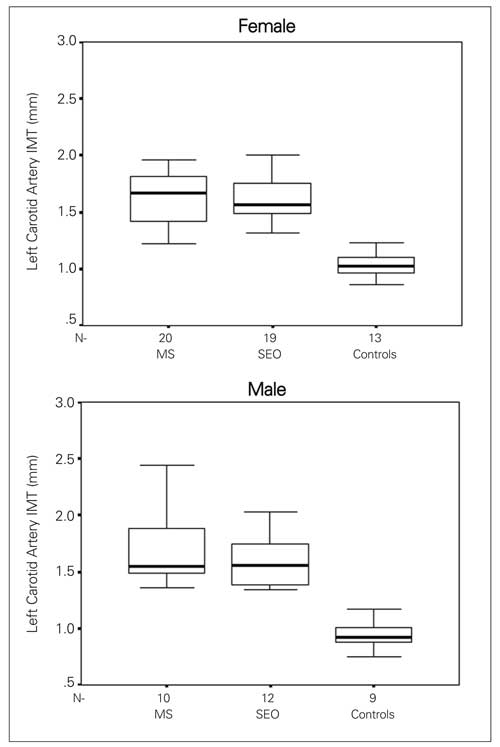
The left carotid artery intima-media thickness (IMT) of the obese groups and controls *Median, 25^th^-75^th^ centiles, minimum and maximum values are denoted.

**Table 1:**
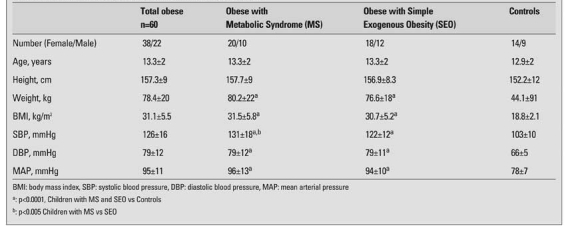
Anthropometric and clinical features of the study population

**2:**
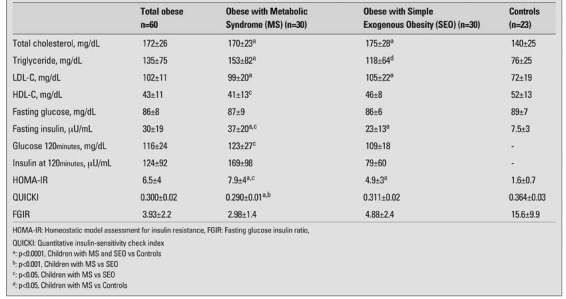
Biochemical and hormonal parameters of the obese children with Metabolic Syndrome, Obese with Simple Exogenous Obesity and in the control groups

**Table 3:**
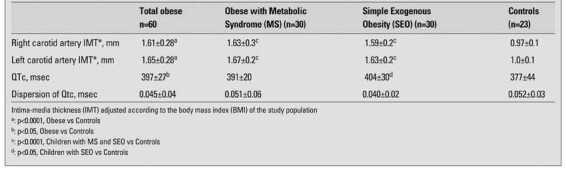
Echocardiographic and electrocardiographic parameters of the obese and control groups (Mean±SD)

**Table 4:**
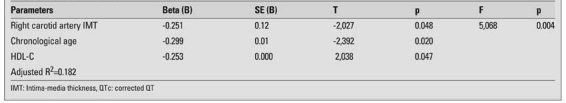
Multivariate linear regression results for QTc

**Table 5:**
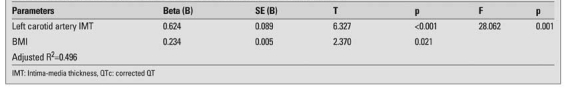
Multivariate linear regression results for right carotid artery intima-media thickness

## DISCUSSION

In the present study, we investigated the effects of clinical and laboratory findings of obese children on the prolongation of QTc and on IMT in both carotid arteries. The prolongation of QTc was mostly affected by chronological age, HDL-C and RCA-IMT in the obese children. Also, lengthening of QTc showed a negative correlation with QUICKI and FGIR in the obese children. There was no statistically significant association between QTc-d and cardiovascular risk factors such as hypertension, insulin resistance and dyslipidemia. We found also a negative correlation between HDL-C and the IMT values of both carotid arteries and BMI in the obese children. QUICKI and FGIR were negatively correlated with IMT of both carotid arteries and also with BMI in the obese children in our study. These results confirmed that insulin resistance associated with decreased serum HDL-C is a risk factor for atherosclerosis and prolongation of QTc, particularly in obese children even at young ages. Also, our results showed that obese children need to undergo cardiological examination, even in the absence of clinical symptoms, because lengthening of QTc can result in sudden death (4). 

The IMT of carotid arteries in the obese children with and without MS were higher than those of the controls. However, there was no difference between the values of carotid artery IMT in the children with and without MS. This implied that obesity alone may be associated with significant impairment of vascular functions. 

In adults, compensatory hyperinsulinemia may lead to persistent QTc lengthening in insulin-resistant patients with obesity and diabetes, even in the absence of autonomic neuropathy (32). Insulin causes QTc prolongation directly or through sympathetic activation. It was shown that resting QTc positively correlated with fasting insulin in the adult population (4). Festa et al (21) found a significant relationship between heart rate-corrected QT interval and carotid atherosclerosis in nondiabetic subjects. These authors also suggested the QTc interval as a marker of undetected atherosclerotic disease. In our study, QTc was found to be significantly affected by RCA-IMT, chronologic age and HDL-C, according to the results of linear regression analysis in obese children with or without MS. We suggest routine electrocardiographic control in obese children, even in those who are free of symptoms. 

Although the relationship between endothelial dysfunction and obesity-related insulin resistance remains unclear, they frequently coexist. Several possible mechanisms between insulin resistance and endothelial dysfunction have been suggested, such as increased production of triglycerides (TG) and impaired endothelium-dependent vasodilatation. Endothelial dysfunction is also well correlated with abdominal obesity (33). Besides, increased LDL-C, lower HDL-C, insulin resistance, hypertension, smoking and obesity are well known cardiovascular risk factors, and the relationship between increased abdominal fat mass and these factors are well documented (14). These risk factors in childhood may induce changes in the arteries contributing to the development of atherosclerosis in adulthood. The measurement of carotid artery IMT is a non-invasive and reliable method for screening the early atherosclerotic changes. Increased carotid artery IMT might be a predictive marker for coronary artery disease. We found that IMT of both carotid arteries became thicker in prepubertal obese children. 

The results of this study also indicate that RCA-IMT can be significantly affected by BMI and LCA-IMT. Sorof et al (34) reported that overweight and hypertensive children have higher carotid IMT than healthy children. In the present study, no significant difference in IMT of the obese children with and without MS was found, while IMT of both carotid arteries were higher than the controls in both obese groups. However, SBP of the obese children with MS was higher than that of the SEO group. These results suggest that obesity is a significant factor for developing atherosclerosis, even if other components of MS such as hypertriglyceridemia and hyperinsulinemia are not present. Oalmann et al (35) have demonstrated that cardiovascular risk factors such as hypertension, obesity, hypercholesterolemia have a role in preclinical atherosclerosis in children. It was reported in the Bogalusa Heart Study that obese children with higher IMT become obese adults, while obese children with normal IMT have normal weight in adulthood (3). 

This study has some limitations. It is a cross-sectional study and the study population consists of children who were referred for obesity. A cross-sectional study design does not prove causality. IMT could be influenced by some hereditary factors, such as HDL-C level, and Turkish people have lower HDL-C levels (36). However, our study showed that HDL-C levels of the controls were higher than those of the obese children with MS. 

In conclusion, significant differences in QTc and IMT of both carotid arteries between obese children and healthy controls were observed in this study. Obese children must be followed closely for early cardiovascular disorders, even in the absence of clinical symptoms.
